# 

**Published:** 1891-11

**Authors:** 


### CONTENTS.

COMMUNICATIONS.

The Thirty-First Annual Meeting of the American Dental Association........ 525
Disease of the Antrum of Highmore........ 549
Dental Ethics........ 555
American Medical Association........ 559
Ohio State Dental Society........ 569

EDITORIAL.

A Peculiar Case........ 570
Dental Meeting........ 572
Bibliographical........ 573
Questions and Answers on Dental Subjects........ 575

